# Criterion validity of the *Postpartum Bonding Questionnaire* using an observer-rated measure of mother-baby bonding as a reference

**DOI:** 10.1590/0102-311XEN080124

**Published:** 2026-01-09

**Authors:** Márcia Leonardi Baldisserotto, Rosane Harter Griep, Mariza Miranda Theme Filha

**Affiliations:** 1 Universidade Federal do Rio de Janeiro, Rio de Janeiro, Brasil.; 2 Escola Nacional de Saúde Pública Sergio Arouca, Fundação Oswaldo Cruz, Rio de Janeiro, Brasil.; 3 Instituto Oswaldo Cruz, Fundação Oswaldo Cruz, Rio de Janeiro, Brasil.

**Keywords:** Surveys and Questionnaires, Postpartum Period, Mothers, Inquéritos e Questionários, Período Pós-Parto, Mães, Encuestas y Cuestionarios, Periodo Posparto, Madres

## Abstract

Currently, there are no studies available on the criterion validity of the *Postpartum Bonding Questionnaire* (PBQ) that use an observer-rated measure of mother-baby bonding as a reference. Thus, this study analyses the PBQ using an observer-rated measure of mother-baby. This is a cross-sectional study of 100 women and their babies who were recruited via convenience sampling. The diagnostic capacity of the PBQ was analyzed using receiver operating characteristic (ROC) curves and the area under the curve (AUC). The ROC curves had AUC values ranging from 0.399 (95%CI: 0.227-0.513, p = 0.114) to 0.559 (95%CI: 0.475-0.642, p = 0.163). There was low criterion validity for the PBQ when using the Infant CARE-Index as a reference. The results are novel and make an important contribution to understanding the criterion validity of the PBQ. However, further research using different methods and populations is needed.

## Abstract (pt)

Atualmente, não há estudos disponíveis sobre a validade de critério do *Postpartum Bonding Questionnaire* (PBQ, *Questionário de Vínculo Pós-Parto*) que utilizem como referência uma medida de vínculo mãe-bebê avaliada pelo observador. Este estudo foi conduzido para preencher essa lacuna. Trata-se de estudo transversal com 100 mulheres e seus bebês recrutados por amostragem de conveniência. A capacidade diagnóstica do PBQ foi analisada utilizando curvas ROC (*receiver operating characteristic*) e a área sob a curva (AUC). As curvas ROC apresentaram valores de AUC variando de 0,399 (IC95%: 0,227-0,513, p = 0,114) a 0,559 (IC95%: 0,475-0,642, p = 0,163). Houve baixa validade de critério para o PBQ ao usar o *Infant CARE-Index* como referência. Esses resultados são inéditos e contribuem significativamente para a compreensão da validade de critério do PBQ. No entanto, pesquisas adicionais com diferentes métodos e populações são necessárias.

## Abstract (es)

Actualmente, no hay estudios disponibles sobre la validez del criterio del *Postpartum Bonding Questionnaire* (PBQ, *Cuestionario de Vínculo Posparto*) que utilicen como referencia una medida de valoración del vínculo madre-bebé realizada por un observador. Por lo tanto, este estudio analiza este aspecto del PBQ utilizando una medida de la relación madre-bebé evaluada por un observador. Se trata de un estudio transversal de 100 mujeres y sus bebés, reclutados mediante muestreo por conveniencia. La capacidad diagnóstica del PBQ se analizó utilizando curvas características de funcionamiento del receptor (ROC) y el área bajo la curva (AUC). Las curvas ROC tuvieron valores AUC que oscilaron entre 0,399 (IC95%: 0,227-0,513, p = 0,114) y 0,559 (IC95%: 0,475-0,642, p = 0,163). La validez del criterio del PBQ fue baja cuando se utilizó el *Infant CARE-Index* como referencia. Estos resultados son novedosos y constituyen una importante contribución a la comprensión de la validez de criterio del PBQ. Sin embargo, es necesario realizar más investigaciones utilizando diferentes métodos y poblaciones.

## Introduction

The mother-baby bond is an emotional relationship that is crucial to the physical and mental well-being of the dyad ^1^ ^,^ ^2^. The mother-baby bond is a challenging construct to measure, and scientific research and clinical practice need valid and reliable instruments to do so ^3^ ^,^ ^4^. Although self-report instruments based on the mother’s perceptions are more widely used, observer-rated measures performed by a qualified observer are considered the most accurate method for assessing the mother-baby bond ^5^ ^,^ ^6^. The lack of observer-related measures may be explained because direct observation methods are more time consuming and expensive, requiring a controlled environment to observe interactions and trained researchers to interpret the results ^5^.

Among the self-reported instruments available for measuring the mother-baby bond, the *Postpartum Bonding Questionnaire* (PBQ) is the most used in several countries, mainly in developed ones ^6^. The PBQ was developed to measure mother-baby bonding disorder and comprises 25 items that are divided into four factors: factor 1 is considered a general factor; factor 2 relates to symptoms of rejection and pathological anger of the mother; factor 3 compromises anxiety symptoms of the mother towards her baby; and factor 4 shows symptoms of potential abuse by the mother ^7^ ^,^ ^8^.

The PBQ has been validated in various countries ^9^ ^,^ ^10^ ^,^ ^11^ ^,^ ^12^, and the one adapted in Brazil showed good psychometric properties of structural validity and reliability. In the Brazilian version, the instrument had 12 items distributed in three factors: factor 1 was composed of five items related to symptoms of lack of pleasure in the mother’s interaction with the baby; factor 2 has 4 items related to the mother’s symptoms of rejection and pathological anger towards the baby; and factor 3 is composed of symptoms of anxiety and risk of maltreatment of mothers in relation to the baby. The Brazilian Portuguese version of the PBQ was based on empirical evidence and theoretical considerations during the validation process. The three dimensions aligned with the theoretical framework of impaired bonding and demonstrated adequate psychometric properties. Thus, the PBQ was refined to include 12 items across these three factors, thereby enhancing its clarity and utility in both research and clinical settings, while effectively assessing mother-baby bonding problems ^13^ ^,^ ^14^.

The PBQ is generally considered a validity and reliable instrument ^6^. However, there is no consensus on the adequacy of its criterion validity ^6^ ^,^ ^15^. Studies have shown that the total score and factor 1 can be used to identify bonding disorder symptoms ^7^ ^,^ ^8^ ^,^ ^12^ ^,^ ^16^. Notwithstanding, for factors 2 and 3, the results regarding their criterion validity are controversial, of which some studies show satisfactory values ^12^ ^,^ ^16^ and others, unsatisfactory ^7^ ^,^ ^8^. Most studies show poor criterion validity regarding factor 4 (incipient abuse) ^16^. Note that these studies have used other self-reported instruments as the criterion measurement ^12^ ^,^ ^16^.

To date, therefore, there is no study on the criterion validity of the PBQ that use an observer-rated measure of mother-baby bonding as a reference. This gap was pointed out in a recent systematic review of parent-reported assessment measures, wherein the authors argue that an observer-rated measure is necessary for verifying the criterion validity of the PBQ ^6^. Moreover, authors have recommended using an instrument involving quite different methods (e.g., self-reported versus observational instruments) as a criterion measure to check the criterion validity of an instrument ^17^.

The Infant CARE-Index (ICI) is one of the observer-rated measurement instruments available ^18^ that assesses the quality of the mother-baby relationship. This has been used in several countries and cultures worldwide and its validity and reliability has been confirmed ^18^ ^,^ ^19^ ^,^ ^20^. For this study, the ICI instrument was chosen over others also because its theoretical conceptualization of mother-infant bonding is very similar to that used in the PBQ ^5^ ^,^ ^7^ ^,^ ^17^.

This study analyses the criterion validity (sensitivity, specificity) of the PBQ using an observer-rated measure of mother-baby bonding as a reference to contribute to an understanding of its psychometric properties. Thus, this study contributes novel information to our understanding of this instrument.

## Method

### Study design, participants, and procedures

This cross-sectional study sampled 100 mothers over 18 years of age who had 2-12-month-old babies assisted at a primary care unit in Rio de Janeiro, Brazil. The sample was recruited via convenience sampling. All eligible participants who agreed to participate answered a structured questionnaire containing questions about their sociodemographic characteristics and the PBQ. Moreover, each mother was filmed with her baby for three to five minutes in a private room structured according to the ICI protocol, and they were invited to interact or play with their children as normal.

### Variables of the questionnaire

(a) Sociodemographic and pregnancy data: mothers’ age, self-reported skin color, marital status, education, paid work, parity, the intentionality of pregnancy, and her satisfaction with it.

(b) PBQ: a self-reported instrument assessing mother-baby bonding disorders ^14^. The Brazilian version used in this study comprises 12 items in a hierarchical structure, with three first-order factors and one overall second-order factor ^13^ ^,^ ^14^. The total score for the Brazilian version of the PBQ is 0-60, with a higher score indicating a higher risk of a mother-baby bonding disorder ^14^.

(c) ICI: this method assesses the quality of interaction between infants and their caregivers, from 0 to 15 months of age. The full ICI protocol was followed in this research. The three to five-minute video recordings of mother-infant interactions were coded by a trained researcher. After this, the researcher assigned a score to each dyad according to interaction patterns rated using seven subscales: three for the caregiver (sensitivity, control, and non-responsiveness) and four for the infant (cooperation, compulsivity, difficulty, and passivity). Next, mother and infant were rated separately on the following seven aspects of the interaction: facial expressions, verbal expression, body position and contact, affect, control, and activity choice ^18^. Based on this assessment, the coder gave an overall score for the quality of the mother-infant dyad interaction ranging from 0 to 14. The higher the score, the better the quality of the relationship. The score is classified into four categories: “sensitive” (score of 11-14), characterized by joy interaction and positive affection; “adequate” (score of 7-10), characterized by satisfactory interaction; “inadequate” (score of 5-6), characterized by limited playfulness and unresolved problems, but with no evidence of hostility; “at risk” (score of < 4), characterized by an evident lack of empathy and a complete failure to perceive the child’s state ^20^.

This protocol was performed by two Latin American coders certified in the ICI method. Any divergent assessments were discussed, and a single score was given for the mother and the infant separately and another score was given for the quality of each mother-infant relationship. The score assigned to the mother, ranging from 0 to 14, was used as criterion reference for this study ^20^.

### Data analysis procedures

Two categorical variables were created from the ICI scores that were given to the mother: those coded “yes” for “very poor bond” had an ICI score of 0-4, and those coded “no” had an ICI score of 5-14; and mothers coded “yes” for “poor bond” had an ICI score of 0-6, and those coded “no” had an ICI score of 7-14. This categorization was based on the cut-off points of the final score categorization of the ICI ^20^.

To compare the groups of mothers classified as having a very poor bond or poor bond, we conducted stratified analyses of the mothers’ sociodemographic and pregnancy characteristics, and the mother-baby bonds were measured using the PBQ. Differences in proportions between the groups were tested using the χ^2^ test and differences in means between the groups were tested using Student’s t-test. The significance level used for both tests was 5%.

The criterion validity ^15^ of the PBQ (total score and per factor) were verified using a receiver operating characteristic (ROC) curve analysis. In this analysis, the area under the curve (AUC), which is an estimator of the general accuracy of a measurement instrument, and its 95% confidence interval (95%CI) and statistical significance were calculated. The AUC values were classified as: > 0.9 (high accuracy), 0.9-0.7 (moderate accuracy), 0.7-0.5 (low accuracy), and < 0.5 (very low accuracy) ^21^. Statistical analyses were performed using R v. 3.5.1 (http://www.r-project.org).

### Ethics

This study was approved by the Ethics Committee of the Sergio Arouca National School of Public Health in Oswaldo Cruz Foundation (ENSP/FIOCRUZ, n. 13810919.5.0000.5240). All participants signed an informed consent form and authorized the use of images recordings. The interviews and filming were conducted in a private room, ensuring the privacy of participants.

## Results

Regarding the maternal characteristics according to the type of mother-baby bonding classified with the ICI, there was a statistically significant difference in the proportion of mothers with a very poor bond in which the mother had only that one child and in which she had more than one child. For the other variables tested, there were no significant differences between the groups (Table 1).

**Table 1:** Sample sociodemographic characteristics and the total score of the *Postpartum Bonding Questionnaire* (PBQ) according to the mother-baby bonding categorized according to the Infant CARE-Index (ICI) (n = 100).

Sample characteristics	Infant CARE-Index					
	n	Very poor bond			Poor bond		
		Yes (%)	No (%)	p-value	Yes (%)	No (%)	p-value
Maternal age (years) [mean±SD]	28.3±6.4	27.6	28.8	0.354	28.3	28.3	0.958
PBQ * [mean±SD]	6.0±1.8	6.8	6.1	0.412	7.1	6.2	0.317
Has paid work				0.135			0.063
Yes	32	31.3	68.7		65.6	34.4	
No	68	47.0	52.0		82.4	17.6	
Skin color				0.748			0.727
White	30	46.7	53.3		73.3	26.7	
Black	18	44.5	55.5		83.3	16.7	
Brown	52	38.5	61.5		76.9	23.1	
Number of children **				0.043			0.157
1	34	55.8	44.1		85.3	14.7	
More than 1	66	34.9	65.1		72.7	27.3	
Schooling level (years)				0.815			0.623
< 8	23	47.8	52.8		78.3	21.7	
8-11	27	40.7	59.3		70.4	29.6	
≥ 12	50	40.0	60.0		80.0	20.0	
Intentionality of pregnancy				0.892			0.603
Intended	48	39.6	60.4		81.2	18.8	
Wanted to wait longer	20	45.0	55.0		75.0	25.0	
Not intended	32	43.8	56.2		71.8	28.2	
Satisfaction with pregnancy				0.966			0.213
Satisfied	63	41.3	58.7		82.5	17.5	
More-or-less satisfied	26	42.3	57.7		69.2	30.8	
Unsatisfied	11	45.4	54.6		63.6	36.4	
Marital status				0.063			0.772
Lives with a partner	76	36.9	63.1		76.3	23.7	
Does not have or live with a partner	24	41.7	58.3		79.2	20.8	
Total	100	42	58	-	78	22	-

SD: standard deviation.

Note: in bold: p < 0.05.

* Baldisserotto et al. ^14^.

** Excluding the baby.

**Figure 1:**
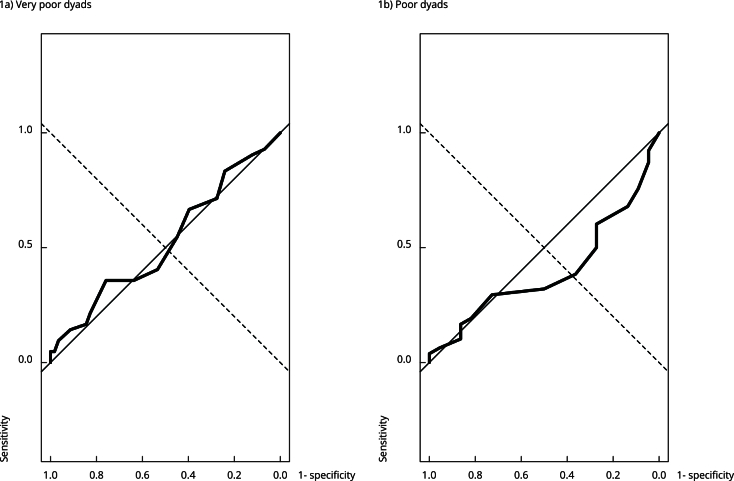
ROC curves for the total *Postpartum Bonding Questionnaire* (PBQ) * score for detection of very poor (upper) and poor (lower) mother-baby bonds, classified based on the Infant CARE-Index (ICI).

The ROC curve analyses of the total score of the PBQ found that the AUCs for identifying mother-baby bonds classified as very poor (AUC = 0.525, 95%CI: 0.409-0.642, p = 0.665) or poor (AUC = 0.399, 95%CI: 0.276-0.513, p = 0.114) based on the ICI were not statistically significant (Figure 1).

* Baldisserotto et al. ^14^.

**Figure 2:**
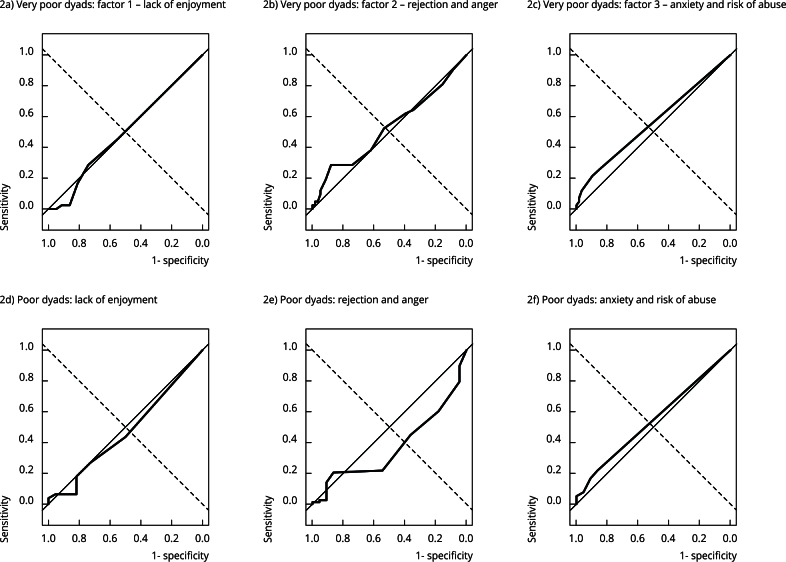
Per-factor ROC curves for the *Postpartum Bonding Questionnaire* (PBQ) *, for detection of mother-baby bonding classified as very poor (upper panels) and poor (lower panels) based on the Infant CARE-Index (ICI).

In the per-factor ROC curve analyses, the AUCs for identifying mother-baby bonds classified as very poor were not statistically significant (F1: AUC = 0.493, 95%CI: 0.388-0.597, p = 0.892; F2: AUC = 0.525, 95%CI: 0.407-0.643, p = 0.677; F3: AUC = 0.559, 95%CI: 0.476-0.642, p = 0.163). For identifying bonds classified as poor, factor 2 had a statistically significant AUC (AUC = 0.371; 95%CI: 0.247-0.495; p = 0.042), factors 1 and 3 did not (F1: AUC = 0.471, 95%CI: 0.343-0.598, p = 0.892; F3: AUC = 0.543, 95%CI: 0.457-0.629, p = 0.323) (Figure 2).

95%CI: 95% confidence interval; AUC: area under the curve. Note: 2a) AUC = 0.493 (95%CI: 0.388-0.597); p-value = 0.892; 2b) AUC = 0.525 (95%CI: 0.407-0.643); p-value = 0.677; 2c) AUC = 0.559 (95%CI: 0.475-0.642); p-value = 0.163; 2d) AUC = 0.471 (95%CI: 0.343-0.598); p-value = 0.651; 2e) AUC = 0.371 (95%CI: 0.247-0.495); p-value = 0.042; 2f) AUC = 0.543 (95%CI: 0.457-0.629); p-value = 0.323. * Baldisserotto et al. ^14^.

## Discussion

This study provides the first available information on the criterion validity of the PBQ, which use an observer-rated measure of mother-baby bonding as a reference. As already mentioned, other studies have used self-reported instruments as a criterion measure ^6^. The results herein indicated low criterion validity for the PBQ when the ICI observer-rated method was used as a criterion measure. In the ROC curve analyses, the AUC values and their associated p-values were not statistically significant. Therefore, it was not possible to establish valid cut-off points for the use of the PBQ to identify very poor and poor mother-child bonds as defined here according to the ICI.

As already mentioned, the studies on criterion validity of the PBQ indicate that the total score and factor 1 have adequate diagnostic ability (sensitivity and specificity) and factor 4 was inadequate ^7^ ^,^ ^8^ ^,^ ^12^ ^,^ ^16^. Factors 2 and 3, on the other hand, show controversial results, with no consensus in literature ^7^ ^,^ ^8^ ^,^ ^12^ ^,^ ^16^. However, the difference in the instruments used as a criterion measure may limits the potential comparability of the results of this study.

The low criterion validity of the PBQ may be a result of the “social desirability and faking good” bias ^22^, which is probably present in any self-reported instrument that measures sensitive constructs ^7^ ^,^ ^9^ ^,^ ^10^. This may be because motherhood and the mother-baby bond are burdened with prejudice and stigma ^23^, leading women to respond intentionally or unintentionally with the goal of social acceptance. In this sense, self-reported instruments for assessing the mother-baby bond are likely to be limited in their ability to detect problems, since the symptoms of child abuse related to more severe manifestations of bonding disorders may be underreported ^7^.

Moreover, the low criterion-validity values of the PBQ may be the result of differences between the mother’s perception of her relationship with her baby and the perception of a third party (the observer). Although other authors have recommended the comparison of different instruments for this type of study ^17^ and the use of observer-rated measures of mother-baby bonding as a reference6, this difference in measurement methods may yield non-comparable results.

Despite making an important contribution to our knowledge concerning the validity of the PBQ, this research has some limitations. First, a convenience sample that included mothers attending a primary healthcare unit was used, which limits the external validity of the findings; thus, caution in extrapolating our findings is required. Therefore, other studies using different populations are needed to corroborate our findings. Another limitation concerns the artificiality of the setting for the filming, which was set up according to the ICI protocol. However, there may have been distortions or a bias in the range of behaviours, because this was an unnatural environment. Studies using other observed-rated methods of assessing the mother-baby bond are necessary to further test the criterion validity of the PBQ. Lastly, because of the characteristics of the population, the PBQ was administered in-person by a trained interviewer, which may have increased the social desirability and faking good bias ^22^.

In conclusion, this study compared the PBQ with an observer-rated measure (ICI) and observed low criterion validity values for the PBQ, which is the most widely used instrument in research on mother-baby bonding. The results are novel and make an important contribution to our understanding of the criterion validity of the PBQ. However, further research with different methods and populations is needed to investigate this psychometrics property of this instrument.

## Data Availability

The research data are available upon request to the corresponding author.
